# Retinal Phosphenes Induced by Transorbital Electrical Stimulation: Influence of Light Adaptation, Electrode Montage, and View Direction

**DOI:** 10.3390/life15050820

**Published:** 2025-05-21

**Authors:** Alexander Hunold, Daniela Ortega, Stefanie Freitag, Dietmar Link, Andrea Antal, Sascha Klee, Jens Haueisen

**Affiliations:** 1 Institute of Biomedical Engineering and Informatics, TU Ilmenau, 98693 Ilmenau, Germany; 2 Research Group in Bioinstrumentation and Clinical Engineering, Universidad de Antioquia, Medellín 050010, Colombia; 3 Department of Neurology, University Medical Center Göttingen, 37075 Göttingen, Germany; 4 Division Biostatistics and Data Science, Department of General Health Studies, Karl Landsteiner University of Health Sciences, 3500 Krems, Austria

**Keywords:** retina, phosphene threshold, tACS, ocular electrical current stimulation

## Abstract

In this study, the perception of phosphenes was used as a surrogate identifier for stimulation sites for use in retinal-degenerative diseases. We aimed to investigate the influence of adaptation, electrode montage, and direction of view on electrically induced phosphenes. We developed a practical methodology to assess non-invasive ocular electrical stimulation, addressing specific areas in the retina. Phosphene thresholds were identified under light and dark adaptation for non-invasive transorbital electrical stimulation. The location and extent characterized the morphology of electrically induced retinal phosphenes for five directions of view and for seven electrode montages in 62 participants. Our results indicated the lowest phosphene thresholds under light adaptation. Cumulative charts of phosphenes visualized the location of phosphene hot spots and their focality for the investigated directions of view and electrode montages. Under consistent light adaptation, we found changes in electrode montage generated stronger changes in the phosphenes’ morphology and distribution. Adjusting the electrodes in the orbital vicinity was more effective than changing the direction of view to shift the phosphene hot spot location to a pathological region to induce neuronal activity there. In this study, we established the first practical methodology to adapt non-invasive transorbital electrical stimulation to address specific areas in the retina.

## 1. Introduction

Non-invasive electrical stimulation of the retina has emerged as a promising technology in clinical research, offering a potential therapeutic intervention for retinal degeneration [1], retinitis pigmentosa [2], and optic neuropathy [3] including glaucoma [4,5,6]. Despite the rather unspecific application of alternating electric current via bilateral electrodes, these pioneering studies have demonstrated the potential of electrical stimulation to restore or enhance visual perception in individuals with retinal degeneration [7]. Investigations into the electrophysiological effects of transorbital electrical stimulation identified the retinal ganglion cells as the primarily responding structures [8]. However, it is currently unclear what would be the optimal stimulation sites, i.e., the number and location of stimulation electrodes, for the different retinal diseases. Also, it is unclear if standard electrode montages or personalized electrode montages would yield increased benefits for the patients [9]. Thus, methods are needed to assess the influence of electrode montages on retinal current flow. Phosphenes (light perceptions) have been used as surrogate markers for actual current flow to better understand the mechanisms of action of transorbital electrical stimulation (toES). The locations of current density hot spots showed a good correlation with the perception of phosphenes in the visual field [10].

Early investigations by Barlow and colleagues [11] shed light on the influence of dark adaptation and light exposure on the phosphene threshold during non-invasive ocular electrical stimulation. Their pioneering study revealed that changes in the adaptation state of the retina significantly affect the perception of electrically induced phosphenes. They demonstrated that phosphene thresholds dropped when the light was extinguished at full light adaptation. The threshold increased exponentially during dark adaptation, settling at a level above the phosphene threshold for light-adapted eyes. However, the electrode montage of Barlow and colleagues [11] consisted of copper disks placed at the forearm and the forehead, which is different from the typical electrode montages used in studies today.

Building upon this foundation, Brindley [12] further explored the site of electrical excitation by positioning stimulation electrodes directly on the eyeball. The reported phosphene pattern originated from the stimulation of the retina by radial current flow. However, positioning stimulation electrodes between the lid and the sclera is a cumbersome procedure and puts a high burden on the patients. Thus, this procedure limits the toES applicability in clinical routine. More-recent non-invasive electrical stimulation of the retina, therefore, applied montages using surface electrodes at the skin surrounding the eyeball [3] or near the orbital cavity [4] at the supraorbital [6] and infraorbital positions [13]. Even though phosphenes were used to determine thresholds for stimulation parameters in these applications, it is unclear how phosphenes are distributed and how light adaptation and direction of view change phosphenes. The knowledge of the factors influencing phosphenes will be helpful in establishing them as a marker in clinical studies [4,6,10,13].

Consequently, we aimed to characterize the distribution of electrically induced retinal phosphenes for the typical orbital electrode montages used in current clinical studies. Moreover, we aimed to characterize the influence of the main directions of view on the phosphene distribution for these electrode montages.

## 2. Methods

We performed two studies, S1 and S2. In study S1, we investigated a practical setting with respect to light or dark adaptation as well as visual contrast for the evaluation of phosphene perception. In study S2, the morphology of phosphenes was evaluated based on individual reports, as introduced by Brindley [12]. All participants in both studies gave written informed consent and confirmed that none of the following contraindications applied: aged less than 18 years or above 65 years; heart disorders or a pacemaker; significantly elevated blood pressure or thyroid hyperfunction; neurological diseases, especially epilepsy or pain syndrome; neurostimulators; psychiatric diseases; current or past substance abuse; pregnancy or breastfeeding; regular medication; ophthalmologic diseases; visual impairments larger than 2 dpt. Only young adults with normal vision participated in these studies to establish baseline data based on a homogeneous sample for initial testing. Nevertheless, we aimed for a balanced sex ratio, minimizing sex-related bias. The study protocols complied with the Declaration of Helsinki and were approved by the Ethics Commission of the University hospital Jena, Germany.

In study S1, we used a cross-over design. A total of 16 healthy (age 28.5 a ± 6.8 a, 7 women) participants tested their phosphene thresholds after light and dark adaptation. A ring electrode (inner/outer diameter: 3/7.5 cm) surrounding the eye [14], and a rectangular electrode (10 cm × 10 cm) over the occiput applied the current output generated by a battery-driven constant-current stimulator (DC-STIMULATOR PLUS, neuroConn GmbH, Ilmenau, Germany). The stimulator was remotely fed with a zero-offset 20 Hz sine wave controlled by a function generator (Agilent 33250A, Keysight Technologies Inc., Santa Rosa, CA, USA) since this waveform resulted in the most intense phosphenes in a study with frontal and occipital stimulation electrodes [15]. The participants adjusted the stimulation intensity on the rotary control of the function generator with a covered display [16].

The setup was completed by goggles, fixating the electrodes, and implementing the adaptation condition. For dark adaptation, the goggles were covered with a sleep mask. For light adaption, the goggles were cut open, so participants perceived the artificial room luminance at 125 cd/m^2^ ± 11 cd/m^2^, measured with a spectroradiometer (Specbos 1201, JETI Technische Instrumente GmbH, Jena, Germany).

**Figure 1:**
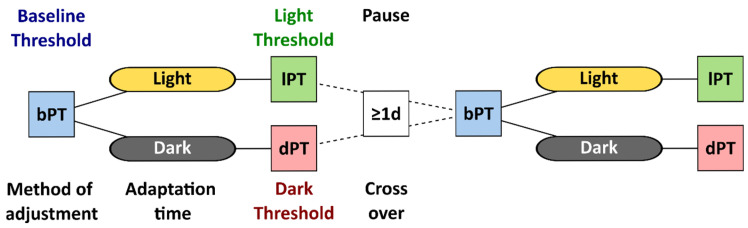
Flowchart of study S1 with the phosphene threshold (PT) determined by the method of adjustment at baseline (bPT), after dark or light adaptation, called light and dark thresholds (lPT and dPT). After a pause of at least one day, participants repeated the session with the baseline and the cross-over adaptation condition.

Under the light-adapted baseline condition, the participants used a method of adjustment to determine their phosphene thresholds. The study personnel averaged the current intensities from ten attempts and determined the reported baseline phosphene threshold. First, a time window of 40 min was granted to ensure steady-state light or dark adaptation [11]. This time window passed in all cases before a second session of phosphene threshold determination, with the method of adjustment performed under light or dark adaptation. Figure 1 visualizes the flow in study S1, where the participants were randomly assigned to the initial adaptation condition, and the session with the cross-over condition was conducted after a pause of at least one day as a wash-out period to minimize implementation bias.

The phosphene thresholds (PTs) under 40 min dark adaptation (dPT) were contrasted to the thresholds at baseline (bPT) and to the threshold after 40 min under light adaptation (lPT).

In study S2, we asked participants (two participants from S1 took part in S2) to immediately report their phosphene perception dependent on the electrode montage and direction of view (DoV). Two groups of 31 (age 28.0 a ± 5.0 a, 14 women) and 31 (age 23.9 a ± 3.6 a, 14 women) healthy participants shared eight participants (one participant took part in the two groups in study S2 and in study S1) and reported their phosphenes by pencil drawings on perimeter charts [12] with an azimuthal angle φ up to 90 °and a polar angle θ.

**Figure 2:**
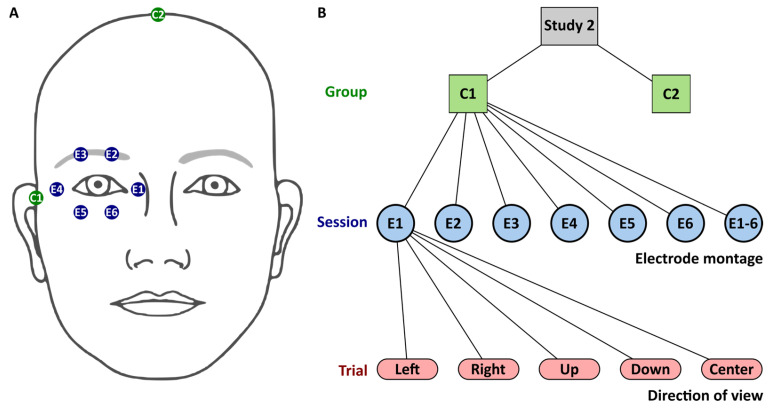
Study S2. (**A**) Stimulation electrodes (E1–E6) surrounding participants’ right eye and counter electrodes (C1 and C2) were located at the right temple in Group 1 (C1) or at the vertex in Group 2 (C2). (**B**): Flowchart with levels for group, session, and trial. Session level comprised the seven electrode montages involving either one electrode, E1 to E6, or all electrodes E1-6. The trial level comprised five directions of view. Sessions and trials were iterated in randomized order.

toES was applied via six silver-silver chloride cup electrodes with a 10 mm diameter surrounding the right eye, as depicted in Figure 2A. The counter electrode, of the same type, was positioned at the temple in Group 1 and at the vertex in Group 2. The cup electrodes were filled with conductive paste (Ten20, Weaver & Co., Aurora, CO, USA).

Participants sat on a chair and placed their heads in a chin–forehead rest. They looked at a black curtain at a distance of 1 m with red fixation targets positioned in the center and at a visual angle of 30 degrees in the directions: left, right, up, and down. A black curtain was chosen because, in pilot experiments, participants more easily described phosphene morphologies when looking at a dark background.

Participants received super-threshold stimulation with 500 µA peak-to-peak (µApp) in each stimulation trial for phosphene detection. The stimulation with a 3 s fade in/fade out and 10 s duration was generated with a multichannel constant-current stimulator (DC-STIMULATOR MC neuroConn GmbH, Ilmenau, Germany).

Each participant performed stimulation sessions with seven different electrode montages. In each session, either one stimulation electrode (E1 to E6) or all stimulation electrodes (E1-6) were active at a time in a randomized order. Each stimulation session comprised 5 trials. In each trial, participants had to gaze in different directions (center, left, right, up, and down) in a randomized order. The direct report of phosphene sensation by participants minimized subjective bias, as it relied on immediate feedback rather than relative subjective interpretation. The flowchart of study 2 is depicted in Figure 2.

**Figure 3:**
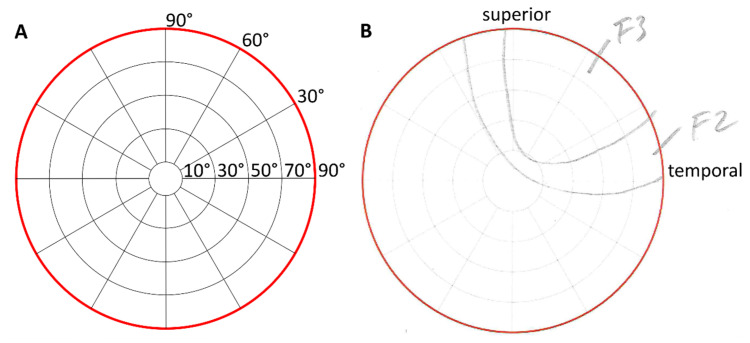
Perimeter charts for phosphene drawings as the template according to Brindley [12] (**A**) and a scan of an original drawing of a participant (**B**) with anatomical directions. In this sample, the participant reported flickering areas with intermediate intensities of grades 2 and 3.

The change in the DoV of 30 degrees in each direction (left, right, up, down) was supported by red fixation targets on a black curtain. Participants kept both their eyes on each fixation target for 10 s while the stimulation was applied to the right eye only. After each stimulation, participants were asked to draw the morphology of the perceived phosphene on the perimeter chart and to rate its characteristics (see Figure 3). Phosphene characteristics were classified as flicker, constant bright, or shade. Each of these categories was rated from 1 to 5, with 1 being just above the limit and 5 being as bright as the sun.

Drawings of phosphene morphologies for each electrode montage and DoV were digitized. Image processing using OpenCV 4.2 in Python 3.7 [17] was used to separate each section in the drawings, and text recognition was applied to identify the categories of the participants’ classification. Drawn regions from all participants per group were superimposed for each electrode montage and DoV. The superimposed regions resulted in a cumulative distribution of perceived phosphenes. The congruence of the reported phosphenes was evaluated based on the intensity and the focality. The intensity increased when the areas drawn by different participants overlapped. The focality measured the spread of congruently reported areas as the fraction of the perimeter chart containing the 75% intensity percentile. The center of mass of the 95% intensity percentile of the cumulative phosphene distribution was characterized by its polar (φ) and azimuthal (θ) coordinates and was used to track the changes in the localization of the perceived phosphene’s hot spot depending on the electrode montage and the DoV. Hot spot locations from the electrode montage E1-6 and the DoV to the center served as the references (ref) to evaluate changes due to different electrode montages and DoVs (test) based on distances (d) in the perimeter chart calculated with the law of cosines according to

$$d=\sqrt{\varphi_{ref}^{2}+\varphi_{test}^{2}-2\varphi_{ref}\varphi_{test}\cos\left(\theta_{test}-\theta_{ref}\right)}$$

## 3. Results

All participants in study S1 reported electrically induced phosphenes and managed to adjust the phosphene thresholds at the current stimulator at the initial baseline condition as well as after light and dark adaptation. The phosphene thresholds of the 16 participants were 132.25 µA ± 73.83 µA, 122.38 µA ± 59.56 µA, and 329.48 µA ± 109.65 µA (mean ± std) at baseline, after light, and after dark adaptation.

**Figure 4:**
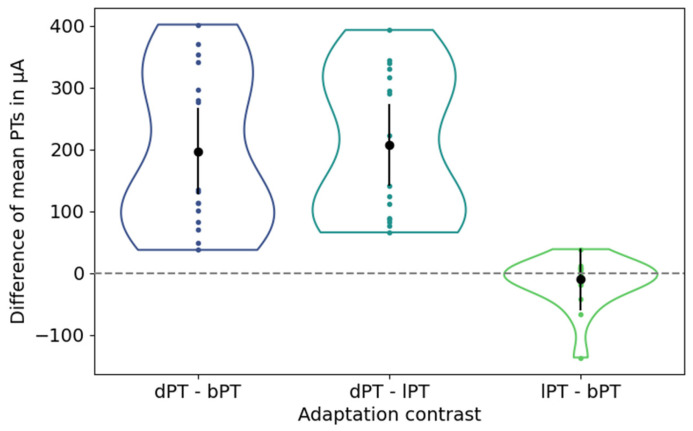
Differences of mean phosphene thresholds (PTs) between adjustments after dark adaptation and at baseline (left), after dark and light adaption (center), and after light adaptation and at baseline (right). Black dots and vertical lines represent the mean differences in mean PTs and their confidence intervals. Each colored dot represents one participant.

The differences in the mean phosphene thresholds between the baseline condition, after dark adaptation, and after light adaptation are depicted in Figure 4.

The mean PT after dark adaptation showed an average difference [confidence interval] of 197.23 µA [127.52 µA, 266.93 µA] and 207.10 µA [141.30 µA, 272.90 µA] to the mean PTs at baseline and after light adaptation, indicating a significant increase in the PTs. In contrast, the mean PTs under the baseline condition and after light adaptation showed an average difference of −9.88 µA with a confidence interval of [−59.90 µA, 40.14 µA], indicating insignificantly lower PTs after light adaptation.

**Figure 5:**
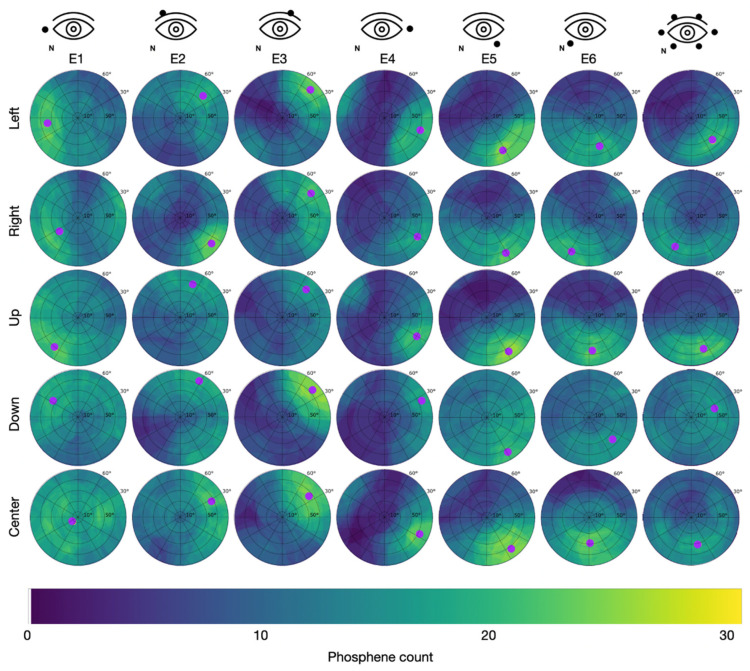
Cumulative phosphene distributions in Group 1 with the counter electrode at the right temple (see C1 in Figure 2) for sessions with different electrode montages in the columns and trials with different directions of view in the rows. Purple dots indicate the center of mass of the 95% intensity percentile and thus represent the hot spot of the cumulative phosphene distribution (color-coded from blue to yellow). The superimposed grid refers to the perimeter chart shown in Figure 3A.

**Figure 6:**
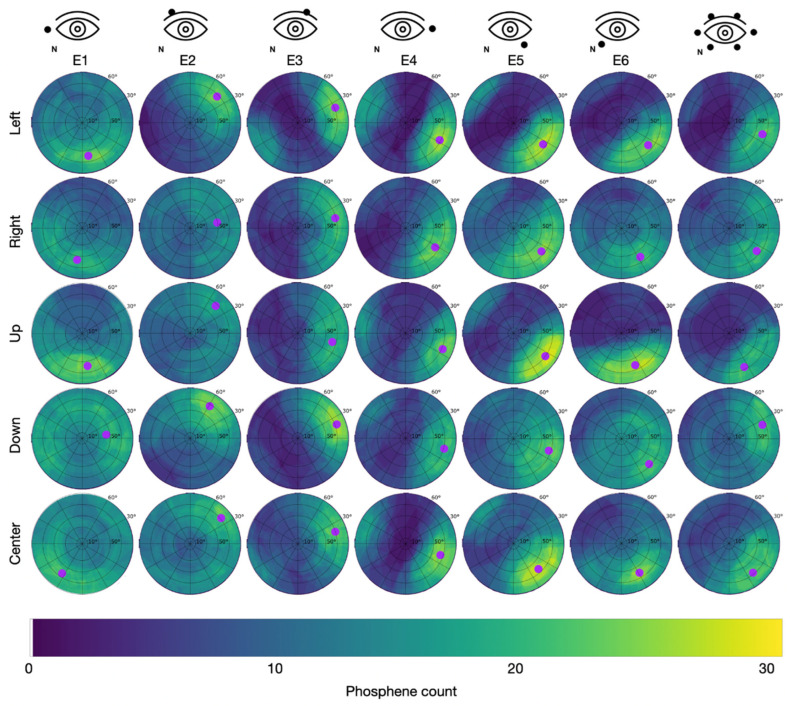
Cumulative phosphene distributions in Group 2 with the counter electrode at the vertex (see C2 in Figure 2) for sessions with different electrode montages in the columns and trials with different directions of view in the rows. Purple dots indicate the center of mass of the 95% intensity percentile of the cumulative phosphene distribution (color-coded from blue to yellow). The superimposed grid refers to the perimeter chart shown in Figure 3A.

Since the PTs were lowest after light adaptation, this condition was used in study S2. In study S2, all participants reported phosphene perceptions in the form of flickering light sensations. The superposition of the 31 individually drawn areas resulted in the cumulative phosphene distributions depicted in Figure 5 for Group 1 (S2), with the counter electrode at the temple, and in Figure 6 for Group 2 (S2), with the counter electrode at the vertex.

The maximal intensity of 31 was reached when the reported phosphene perceptions from all participants incorporated the same spot on the perimeter chart.

Table 1 and Table 2 report the minimal, mean, and maximal values of the cumulative phosphene distributions for each condition introduced in Figure 2, with respect to the electrode montage, DoV, and position of the counter electrode (Group 1 and Group 2 in study S2).

In Group 1 (counter electrode at the temple), the maximum of 27 consistently reported phosphenes occurred for E3 while looking down with the hot spot at φ = 80.4 °, θ = 43.4 ° and for E5 while looking up with the hot spot at φ = 74.7 °, θ = 304.9 °. Across all stimulation sessions, the maximal phosphene count from Group 1 was 22.3 ± 2.5 (mean ± std). The overall average phosphene count in Group 1 was 10.4.

In Group 2 (counter electrode at the vertex), the maximum of 29 phosphenes was consistently reported for E5 while looking left with the hot spot at φ = 66.0 °, θ = 325.2 °, looking up with the hot spot at φ = 71.1 °, θ = 326.8 °, and looking to the center with the hot spot at φ = 63.6 °, θ = 316.9 °. Across all stimulation sessions, the maximal phosphene count from Group 2 was 24.5 ± 2.7 (mean ± std). The overall average of the phosphene count in Group 2 was 9.8.

**Figure 7:**
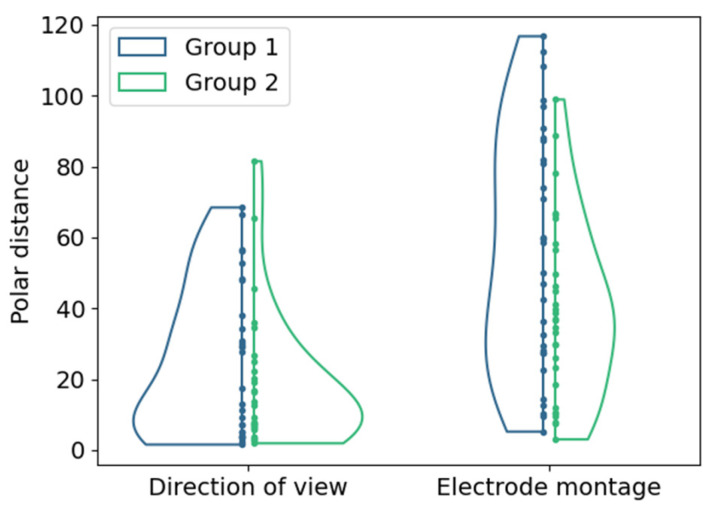
Distributions of polar distances between hot spot locations for both groups (color-coded) with different return electrode positions for (left) changes in DoV with the view to the center as reference; (right) changes in electrode montage with configuration E1-6 as reference. Points represent single samples.

The changes in the location of hot spots as the center of mass of the 95% intensity percentile in the cumulative phosphene distributions were evaluated based on the polar distances (d) shown in Figure 7.

The polar distances in the perimeter chart were 25.4 ± 21.3 and 18.4 ± 18.9 (mean ± std) in Group 1 and Group 2 due to the changes in the DoV. The changes in electrode montages resulted in polar distances between the hot spots in the phosphene counts of 56.0 ± 33.7 and 39.0 ± 24.7 (mean ± std) in Group 1 and Group 2.

**Figure 8:**
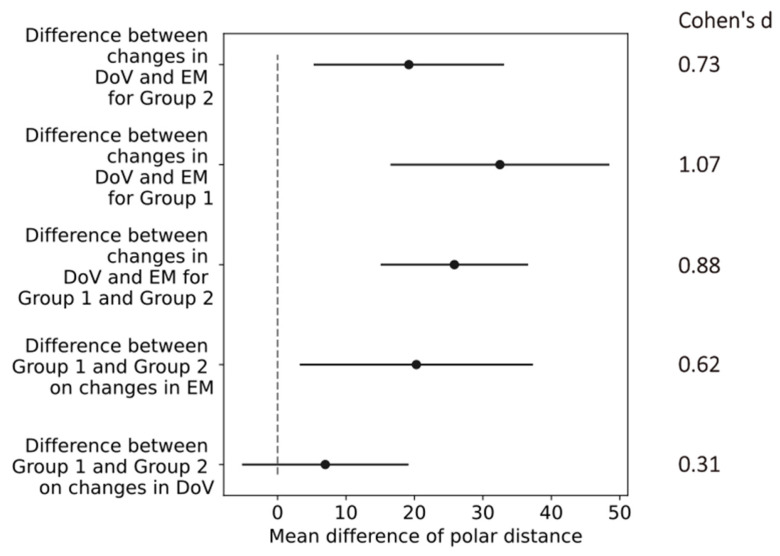
Confidence intervals (black horizontal lines) of difference in mean values (black dots) in polar distances for differences between conditions of return electrode, electrode montages (EMs), and direction of view (DoV) as labeled on the *y*-axis. The gray dashed line marks zero differences. Cohen’s d values on the right indicate effect sizes of comparisons.

The confidence intervals of the differences in the mean values in the polar distances indicated a small effect of the return electrode location (difference between Group 1 and Group 2) on the changes in the DoV with a Cohen’s d of 0.31. All other changes in the return electrode, electrode montages, and directions of view indicated at least medium effects, as shown in Figure 8. The most prominent difference of 30.6 [15.4, 45.86] (mean, [confidence interval]) occurred in Group 1 between the changes in the DoV and the electrode montage, indicating a large effect size with a Cohen’s d of 1.07.

Table 3 reports the area percentage of the perimeter chart containing the 75% intensity percentile of the cumulative phosphene distributions as a focality measure for each condition of the electrode montage, DoV, and position of the counter electrode. The highest focality with the minimal area percentage of 5% containing the 75th intensity percentile occurred for the stimulation at E6 with the counter electrode at the vertex (Group 2) when the participant looked at the center. The lowest focality with a maximal area percentage of 45% containing the 75% intensity percentile occurred for the stimulation at E1 with the counter electrode at the vertex (Group 2) when the participants looked down.

## 4. Discussion

In this study, we established a novel practical methodology of assessing non-invasive transorbital electrical stimulation (toES) addressing specific areas in the retina. The results highlighted the recording of phosphene thresholds under light and dark adaptation for non-invasive toES with electrodes positioned around the orbit. The lowest PTs occurred under light adaptation. The present studies characterized the morphology of electrically induced retinal phosphenes based on their location and extent, depending on the direction of view and the stimulation electrode position. To the best of our knowledge, for the first time, our results demonstrate the changes in the location of phosphene hot spots and their focality depending on the stimulating electrode montage and the direction of view.

Our setup in study S1 ensured well-controlled adaptation conditions and a homogeneous electrical stimulation [18]. We determined that the phosphene threshold at light adaptation was below the threshold at dark adaptation. Our thresholds are considerably lower than the thresholds of 328,9 µA ± 26.4 µA under light and 422.1 µA ± 15.6 µA after dark adaptation found by Barlow et al. [11]. This may be due to the different electrode montages involving a 3 cm diameter stimulation electrode on the forehead and a distant return electrode on the forearm in the study by Barlow et al. [11]. Based on recent studies [4,13], we utilized surface electrodes applied to the skin around the eyeball, where our ring electrode surrounding the orbital cavity together with the occipital return electrode more homogenously stimulated the retina.

Since we determined the lowest phosphene thresholds under light adaptation, we illuminated the laboratory for study S2 purely artificially, ensuring consistent ambient lighting for light adaptation for all experiments.

The morphology of the reported phosphenes in study S2 changed with both the electrode montage, similarly as reported by Brindley [12], and the DoV. Changes in the electrode montage resulted in the most prominent changes in the cumulative phosphene distributions, as the hot spot (center of mass of the 99th percentile) location differed by an average polar distance of 56. This influence was more pronounced with the return electrode close to the stimulation at the right temple (Group 1) as compared to the return electrode at the vertex (Group 2), where the average polar distance was 39. Compared to the electrode montages, the effect of changes in the DoV on the hot spot location was less, with average polar distances of 25.4 (Group 1) and 18 (Group 2). Consequently, adapting the electrode location in the orbital vicinity may be more effective than changing the direction of view to shift the phosphene hot spot location. Directing the hot spot location to functionally impaired and structurally degenerated areas [5] potentially supports the initiation of neuronal activity in a focal pathological region [19].

A detailed study of the effects of alternating-current stimulation on a resected retina of a rabbit model revealed spatially distinct stimulation effects on retinal neurons [19]. In humans, we observed effects of ocular electrical stimulation on the photopic negative response (PhNR) [8] indicating increased activity in retinal ganglion cells (RGCs). This supports the hypothesis that phosphenes are primarily of retinal origin, not cortical [20], aligning with the goal of targeting retinal ganglion cells for therapeutic purposes. The role of phosphenes in retinal degeneration and optic neuropathy is significant, as they serve as functional markers of neuronal activation in the visual system. This is particularly relevant for clinical applications aimed at vision restoration. Prior work supports the notion that optimizing electrode placement and stimulation parameters for transorbital electrical stimulation (toES) can enhance visual function in patients with damaged optic nerves or retinal degeneration [10].

These observed effects on retinal physiology fit well into the ocular luminescence model. Accordingly, the increased RGC activity drives higher levels of reactive oxygen and nitrogen species, which act upon the release of biophotons that can appear as phosphenes [21]. When considering this model in conjunction with intrinsically photosensitive retinal ganglion cells (ipRGCs) [22,23], the reaction of ipRGCs to single photons [24] can explain the phosphene perception under transorbital electrical stimulation.

In prior research, we showed a strong spatial correlation between low PTs and high-current-density areas on the retina [10]. Consequently, perceived phosphenes can be used as a tool to estimate real current flow in the human eye and validate current flow modeling. However, as with any subjective method, compliance of the participants is required for reporting phosphenes, which is a limitation of our studies. Even though phosphene perception can be spatially resolved [25], their description lacks spatial fidelity [26] and introduces ambiguity [27] and variability due to task learning, mental fatigue, or non-compliance [28], in comparison to the perception of light flashes in perimetry. Here, we sought to address this limitation by supporting the description of participants’ perception with phosphene categories and a perimeter chart [25] and interpreted cumulated responses. As per design, the studies included young, healthy participants, which resulted in recruitment bias. Nevertheless, this demographic was chosen to ensure a homogeneous sample for initial testing. In patients with retinal conditions, phosphene perception might be impaired and require higher current intensities [29], whereas patients with disorders affecting higher-order visual processing may not reliably interpret phosphenes [26]. Future work will include older participants to address this limitation and participants with various diseases to broaden the applicability of the findings. The consequent randomization of all study sessions minimized the implementation bias potentially introduced by the operators, performing the sessions with the different participant groups. The direct report of sensation by the participants minimized subjective bias, as it relied on immediate feedback rather than subjective interpretation. Here, we show the ability to shift phosphene hot spots with bipolar toES. In future work, we propose using current flow modeling to select optimal electrode montages and steer the current density to target specific areas of individual pathologies. Moreover, it may be beneficial to consider extending the approach in the current study and superimposing several stimulation channels. This can allow for the use of more degrees of freedom to direct phosphene hot spots.

To the best of our knowledge, our results establish for the first time a practical methodology to adapt non-invasive transorbital electrical stimulation to address specific areas of the retina. Preceding research highlights the therapeutic potential of non-invasive transorbital electrical stimulation in addressing electrophysiological retinal pathologies [7]. We systematically highlight the beneficial adaptation to light in perceiving phosphenes and the potential to shift perceived phosphene hot spots by adapting the stimulating electrode montage. From our perspective, the adaptation of non-invasive transorbital electrical stimulation has great potential to drive advancements applicable in clinical interventions by shifting phosphene hot spots to individual regions with functionally impaired (but not dead) ganglion cells and thus fostering individualization to improve the visual outcomes and quality of life of individuals affected by retinal degeneration [1,2] and optic neuropathy [3,4,5,6].

## Figures and Tables

**Table 1:** Group 1’s minimal, mean, and maximal values (separated by vertical bar) of the cumulative phosphene distributions for different electrode montages (see sessions in Figure 2B: E1, …, E6 and E1-6) in the columns and directions of view (see trials in Figure 2B) in the rows.

	E1	E2	E3	E4	E5	E6	E1-6
Left—Min|Mean|Max	6|13|24	5|12|20	1|10|24	1|6|21	2|8|26	3|10|21	1|8|22
Right—Min|Mean|Max	5|14|23	3|9|25	4|10|23	2|8|20	3|10|24	4|10|21	5|11|19
Up—Min|Mean|Max	7|15|23	5|11|19	3|9|18	3|8|22	1|7|27	2|10|23	3|8|25
Down—Min|Mean|Max	7|15|21	2|13|20	2|7|27	2|7|19	8|14|21	7|11|18	8|13|19
Center—Min|Mean|Max	11|17|23	5|13|23	2|9|25	0|6|25	3|10|26	1|13|24	4|11|20

**Table 2:** Group 2’s minimal, mean, and maximal values (separated by vertical bar) of the cumulative phosphene distributions for different electrode montages (see sessions in Figure 2B: E1, …, E6 and E1-6) in the columns and directions of view (see trials in Figure 2B) in the rows.

	E1	E2	E3	E4	E5	E6	E1-6
Left—Min|Mean|Max	7|14|25	1|9|26	1|7|26	0|8|28	1|6|29	1|6|26	1|6|25
Right—Min|Mean|Max	5|12|21	6|12|18	1|7|23	1|8|26	3|11|27	3|11|22	2|12|22
Up—Min|Mean|Max	6|13|25	6|12|18	2|7|22	3|7|25	2|7|29	1|6|28	3|6|23
Down—Min|Mean|Max	4|16|22	3|11|25	1|6|27	3|8|22	4|12|25	5|14|23	4|11|23
Center—Min|Mean|Max	10|15|23	7|14|24	4|9|25	1|8|26	4|11|29	3|11|28	3|9|23

**Table 3:** Focality as the area percentage of the perimeter chart containing the 75th intensity percentile of the cumulative phosphene distributions for Group 1 and Group 2 (separated by vertical bar) for different electrode montages (see sessions in Figure 2B: E1, …, E6 and E1-6) in the columns and directions of view (see trials in Figure 2B) in the rows.

	E1	E2	E3	E4	E5	E6	E1-6
Left—Group 1|Group 2	21|13	17|9	10|11	24|8	15|14	22|16	15|10
Right—Group 1|Group 2	20|26	10|30	18|15	9|13	8|15	20|17	22|20
Up—Group 1|Group 2	24|20	24|28	17|19	11|9	10|15	18|17	15|14
Down—Group 1|Group 2	43|45	25|16	13|12	10|17	32|18	27|17	19|15
Center—Group 1|Group 2	38|25	19|11	21|12	8|12	19|16	20|5	14|22

## Data Availability

The data in this paper will be made accessible after its publication for non-commercial academic projects that have a legitimate research topic and a clearly stated hypothesis. In the event that the application is accepted, researchers will be asked to have the study approved by their institution’s ethics board. The authors will subsequently provide the datasets via a safe data transfer system.
